# Virus-induced gene silencing of the RPC5-like subunit of RNA polymerase III caused pleiotropic effects in *Nicotiana benthamiana*

**DOI:** 10.1038/srep27785

**Published:** 2016-06-10

**Authors:** Lev G. Nemchinov, Alexander M. Boutanaev, Olga A. Postnikova

**Affiliations:** 1 USDA/ARS, Beltsville Agricultural Research Center, Molecular Plant Pathology Laboratory, Beltsville MD 20705, USA; 2 Institute of Basic Biological Problems, Russian Academy of Sciences, 2 Institute Street, Pushchino, Moscow Region, 142292, Russia

## Abstract

In eukaryotic cells, RNA polymerase III is highly conserved and transcribes housekeeping genes such as ribosomal 5S rRNA, tRNA and other small RNAs. The RPC5-like subunit is one of the 17 subunits forming RNAPIII and its exact functional roles in the transcription are poorly understood. In this work, we report that virus-induced gene silencing of transcripts encoding a putative RPC5-like subunit of the RNA Polymerase III in a model species *Nicotiana benthamiana* had pleiotropic effects, including but not limited to severe dwarfing appearance, chlorosis, nearly complete reduction of internodes and abnormal leaf shape. Using transcriptomic analysis, we identified genes and pathways affected by RPC5 silencing and thus presumably related to the cellular roles of the subunit as well as to the downstream cascade of reactions in response to partial loss of RNA Polymerase III function. Our results suggest that silencing of the RPC5L in *N. benthamiana* disrupted not only functions commonly associated with the core RNA Polymerase III transcripts, but also more diverse cellular processes, including responses to stress. We believe this is the first demonstration that activity of the RPC5 subunit is critical for proper functionality of RNA Polymerase III and normal plant development.

The eukaryotic RNA polymerase III (RNAP III) transcribes key genes involved in protein synthesis, ribosome biogenesis and RNA processing: transfer RNAs (tRNA), 5S ribosomal RNA (rRNA), the spliceosomal U6 small nuclear RNA (U6 snRNA), and the signal recognition particle 7SL RNA^1^. In yeast and human cells RNAP III contains 17 subunits: 12 of them are similar to the core of RNA polymerases I and II and the other five are RNAP III-specific^2^,^3^. The RPC5 subunit in human cells associates with the RPC4 subunit and may be in the proximity to the promoter^4^. It was suggested previously that RPC5 may be required for the RNAP III transcription process^4^. The counterpart of RPC5 in the *Saccharomyces cerevisiae* RNA polymerase III complex is the C37 subunit. The human RPC5 is 32% identical to the *Drosophila* SIN protein (SXL interactor) that interacts specifically with SXL *(sex lethal* protein) in a yeast two-hybrid assay and is considered to be the *Drosophila* homologue of the RPC5^5^. In plants, *Arabidopsis thaliana* SIN-like protein At5G49530 is ~40–85% identical to the proteins described in GenBank as DNA-directed RNA polymerase III subunit RPC5-like (RPC5L). To our knowledge, except for the numerous nucleotide sequences of the predicted RPC5-like subunit in plant species, no further information or gene knockout data are currently available to elucidate the role of the subunit in plant development and responses to the environment.

In this work, an orthologous RPC5 nucleotide sequence from the model species *Nicotiana benthamiana* was used in a virus-induced gene silencing assay (VIGS) to obtain phenotypes with reduced expression of the gene. VIGS is a transcript suppression technique for functional characterization of plant genes^6^. *N. benthamiana* plants with the silenced RPC5L gene were severely dwarfed, had shorter internodes, abnormal leaf shape and defective flowers. Global transcription profiling of the VIGS plants identified diverse genes and pathways affected by reduced expression of RPC5. Based on the transcriptome analysis, silencing of RPC5 has pleiotropic effects and diverse biological roles of the RPC5 subunit in the overall plant development were revealed.

## Results

### Identification of the RCP5L gene

**Figure 1:**
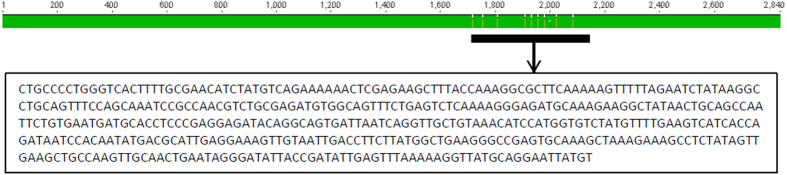
Position of the VIGS fragment (black fill) in the RPC5L gene of *Nicotiana benthamiana* (Niben101Scf03693g06001.1, shown in green). Alignment made with Geneious 8.1.7.

*Arabidopsis thaliana* gene At5G49530 was identified by EST-driven profiling as presumably associated with defense responses^7^. The gene is described in The Arabidopsis Information Resource (TAIR) as a SIN-like family protein containing DNA-directed RNA polymerase III subunit RPC5 domain and possessing RNA polymerase III activity. A tblastn search of the NCBI database with the translated gene product of the At5G49530 as the query sequence resulted in a number of hits with orthologs of the RPC5L in different plant species, including *Nicotiana* species. PCR with primers, designed to amplify the orthologous sequence from *N. benthamiana* (Supplementary Table S1) produced a 446-bp product that was cloned into the TOPO II vector and sequenced (Fig. 1). A blastx search of this sequence against the NCBI nr database, which contains non-redundant sequences from GenBank and other databases such as Refseq, PDB, SwissProt, PIR and PRF, found almost exclusively matches with the RPC5-like subunit in different plant species. Characteristically, that *N. benthamiana* is absent from this list. However, the sequenced fragment was highly homologous to the *N. benthamiana* sequences annotated as RPC5L at Sol Genomics Network, a clade-oriented database dedicated to the biology of the Solanaceae family (https://solgenomics.net/). We thus concluded that the amplified fragment used in this study derived from the RPC5L gene of *N. benthamiana*.

### Phenotypic changes in *N. benthamiana* plants with silenced RPC5L

**Figure 2:**
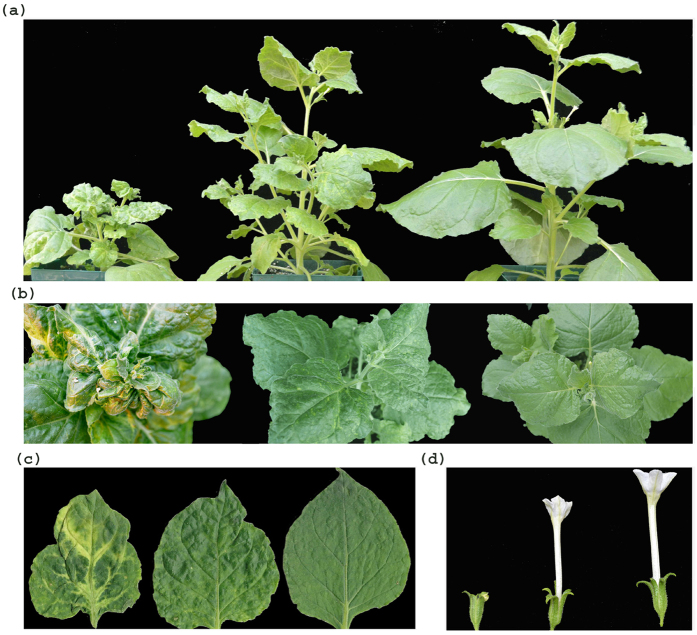
Phenotypical changes caused by virus-induced gene silencing of the RPC5L in *Nicotiana benthamiana* plants. Each panel (**a** through **d**), left to right: VIGS plants; PVX-infected plants; uninfected control.

**Figure 3:**
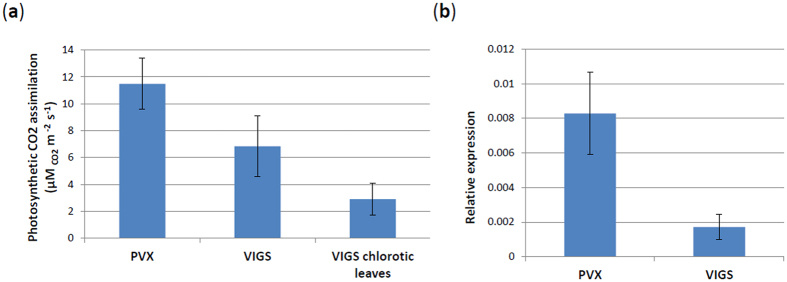
(**a**) The rate of photosynthesis in VIGS plants. (**b**) Expression of the RPC5L gene in PVX-infected and VIGS plants as determined by quantitative real-time PCR.

**Figure 4:**
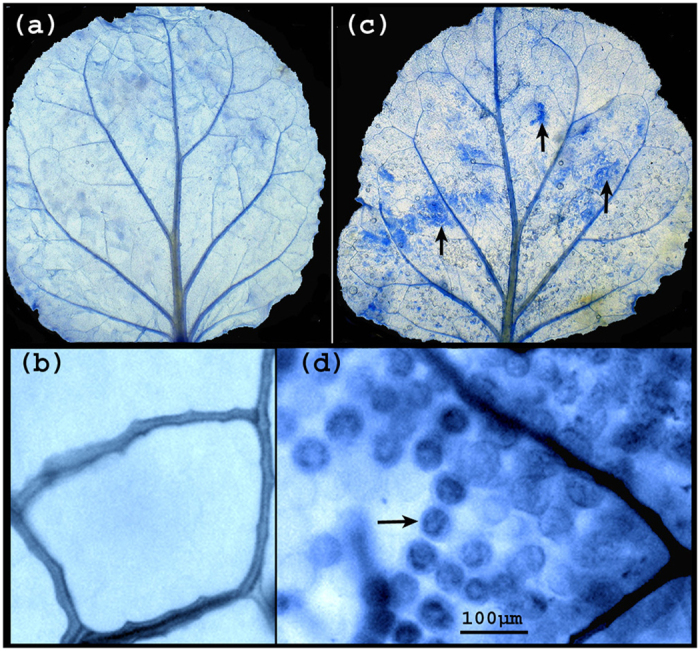
Leaves of *N. bentamiana* plants stained with trypan-blue that specifically stains dead cells. (**a**,**b**) – leaves of the plants infected with PVX alone; (**c**,**d**) leaves from the plants infected with PVX/RPC5L. Arrows indicate regions of localized cell death.

**Figure 5:**
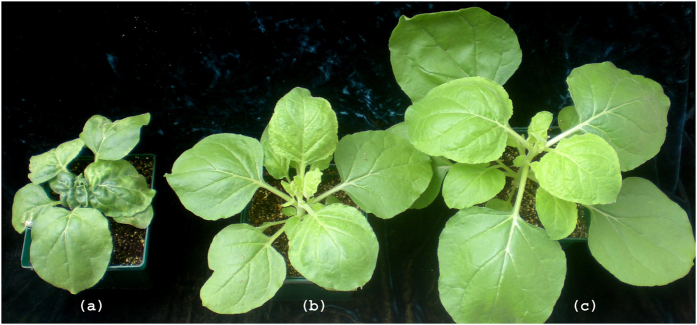
Phenotypes of *N*. *benthamiana* plants infected with *Tobacco rattle virus* (TRV)-based vector carrying RPC5L fragment. (**a**) VIGS plant; (**b**) plant infected with TRV alone; (**c**) uninfected control plant.

Inoculation of *N. benthamiana* plants with a PVX vector carrying the RPC5L sequence triggered unusual phenotypes apparent about three weeks post inoculation (pi). Plants were severely dwarfed with short internode lengths, leaves were abnormally shaped with chlorosis and vein clearing. Flowers were also abnormal, with degenerated short petals (Fig. 2). The chlorotic leaves of the VIGS plants had significantly lower photosynthetic rates than those infected by PVX-wild type (PVX-WT), (Fig. 3a). The phenotypic changes were observed for at least three passages from the original plants inoculated by *in vitro*-produced RNA transcripts. VIGS plants were able to produce viable seeds that grew into normal plants. The phenotypes slightly varied seasonally, being more severe during the fall-winter period than during spring-summer time. Quantitative real-time PCR (qPCR) conducted with a set of primers specific for the RPC5L but located outside of the cloned sequence to prevent amplification from viral RNA (Supplementary Table S1), showed that expression of the RPCL5 gene was inhibited in VIGS plants (Fig. 3b). When random symptomatic leaves from VIGS plants were stained with trypan blue to reveal possible occurrence of localized cell death due to RPC5L silencing, some leaves appeared to have characteristic blue spots consisting of dead cells that absorbed the stain (Fig. 4). To further confirm that the phenotypes observed were not a result of any changes associated with the increased infectivity of recombinant PVX, the same sequence was cloned into a different plant virus vector, pYL156. This vector is based on the *Tobacco rattle virus*, the type species of the genus *Tobravirus* that contains two separately packaged genomes^8^. Two weeks post inoculation similar symptoms started to develop on plants infected with TRV/RPC5L construct (Fig. 5), thus proving that the cause of the abnormal phenotypes is associated with the insert rather than with the virus-vector. qPCR showed that expression of the RPC5L gene in the TRV/RPC5L-infected *N. benthamiana* plants was reduced (not shown).

### RNA-sequencing and transcriptome profiling of VIGS plants

To uncover molecular mechanisms behind the complex phenotypic effects in VIGS plants, we used RNA sequencing to profile the transcriptome.

#### Metrics of RNA-Seq data, sense and antisense activity and RPC5L transcripts

883, 030, 181 of short reads were obtained from 16 strand-specific libraries. The reads were mapped separately in forward and reverse orientation using the RNAseq analysis module in the CLC Genomic Workbench 8.0.2 (Qiagen). The average percentage of the total reads mapped to the reference transcriptome of *Nicotiana benthamiana* and reference genome of *Potato virus X* from the control, PVX-WT and VIGS libraries is shown in Table 1.

An alignment of the 446 nt cloned insert with the draft transcriptome sequence of *N. benthamiana* resulted in the identification of two RPC5L-related transcripts, Niben101Scf03072g06004 and Niben101Scf03693g06001. The identity between them on the nucleotide level was 81.9%, and the alignment had many SNPs, which may indicate that they represent homologous but nevertheless different genes. The RPC5L fragment used for VIGS was 96.8% identical to Niben101Scf03072g06004 and 95.1% identical to Niben101Scf03693g06001. Both mRNAs were silenced, although the down-regulation of the transcripts with the higher homology was greater (expression of the Niben101Scf03072g06004 is shown in Fig. 3).

#### Identification of VIGS-associated transcripts and their functional characterization

**Figure 6:**
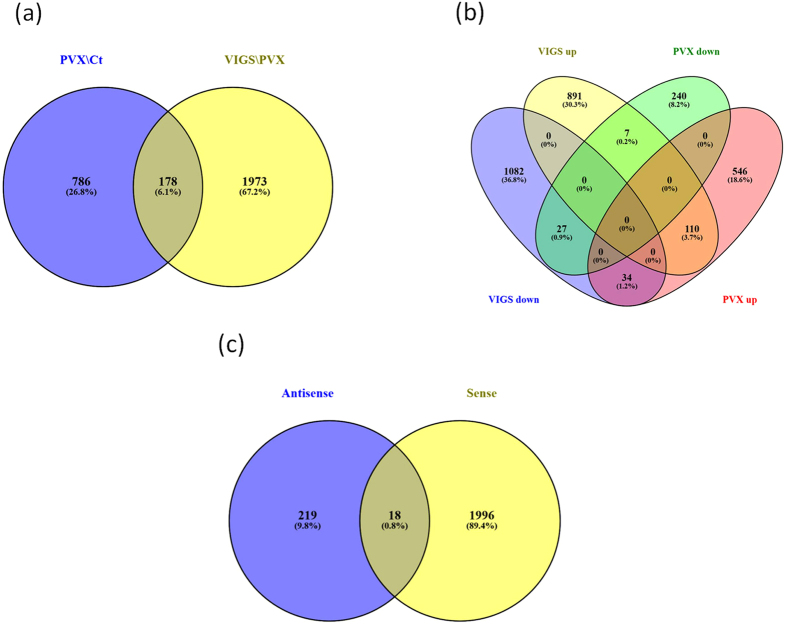
(**a**,**b**) Venn diagrams depicting the numbers of statistically significant DEGs (>2-fold) between PVX-infected and VIGS plants. (**c**) Comparison between the numbers of DEGs identified after mapping the reads in sense and antisense orientations.

Transcriptome analysis led to the identification of a number of differentially expressed genes (DEGs), associated both with PVX-WT infection and VIGS (Fig. 6a,b). DEGs unique for VIGS plants were depicted against the background of a wild type PVX infection. A shared set of 178 genes was found when virus-related genes (PVX\Ct) were compared to VIGS-associated genes (VIGS\PVX). Genes with congruent expression (137 out of 178) were excluded from the analysis to obtain the VIGS-specific set of genes (1973 unique DEGs + 41 discordant common DEGs = 2014 genes). Total number of the DEGs in VIGS plants and their broad biological roles demonstrate a wide-ranging influence of RPC5L silencing on the gene expression pattern (Supplementary Table 2).

Analysis of transcription activity on the antisense strand of protein coding RNA revealed 237 DE regions; 18 of them overlapped with sense DEGs identified in response to VIGS and five had discordant gene expression (Fig. 6c). Antisense expression can affect transcription of the mRNA and therefore carry out many regulatory roles^9^. Up-regulation of some antisense transcripts, annotated via their sense-strand counterpart, may reflect undergoing VIGS processes directed against target host mRNA. For example, antisense transcripts related to the protein argonaute 1 (Niben101Scf07678g07011.1) could take part in the regulation of the respective gene, whose protein product is required for RNA-mediated gene silencing (Supplementary Table S2).

**Figure 7:**
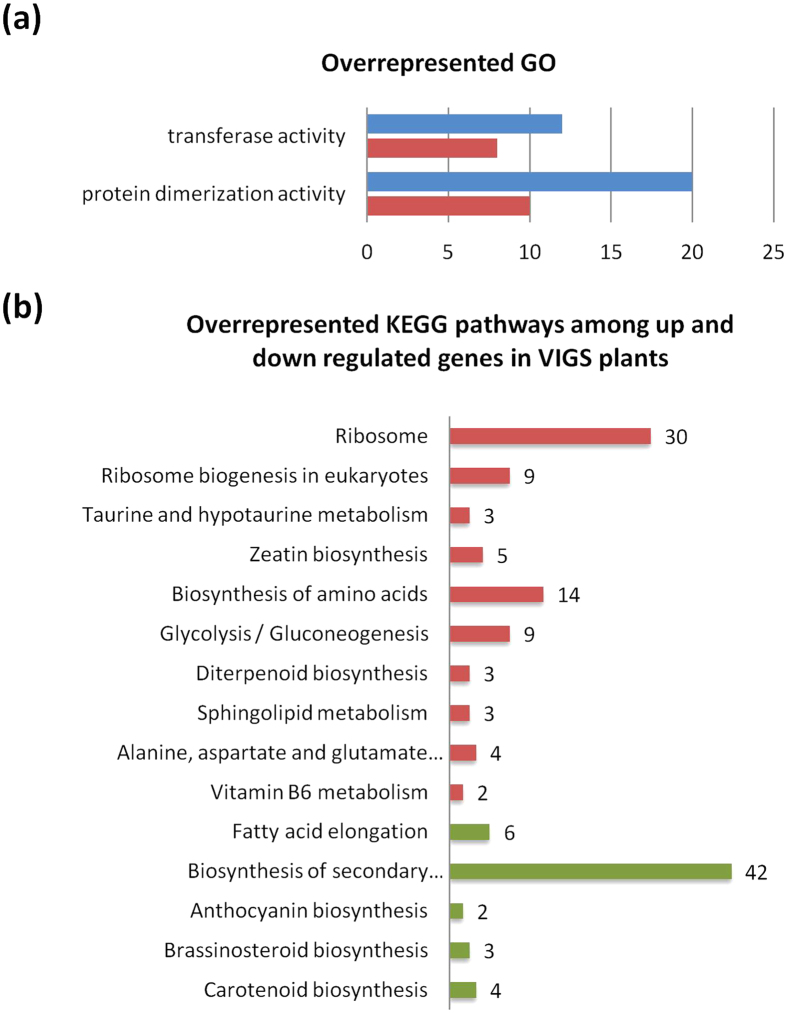
Enrichment analysis of differentially expressed genes using Gene Ontology and KEGG databases. (**a**) GO categories overrepresented among up and down-regulated gene sets in VIGS plants. Red color: up-regulated gene sets in each category; blue color: down-regulated gene sets in each GO category. (**b**) KEGG pathways overrepresented in VIGS plants. Red color: number of up-regulated genes in pathways; green color: number of down-regulated genes in pathways.

Gene Ontology (GO) enrichment analysis discovered groups of genes that were the most affected and responsive to silencing of RCPL5^10^. Using the AgriGO toolkit^11^ and available *N. benthamiana* GO terms (http://solcyc.solgenomics.net/BENTH/class-tree?object=Gene-Ontology-Terms), we found categories overrepresented among down and up-regulated gene sets (Fig. 7a). Down-regulation of genes in the GO term *protein dimerization activity* indicates that protein-protein interactions essential for all biological processes, are disrupted in VIGS plants as a result of reduced RCP5L expression. A number of transcription factors (TFs) in this GO term that regulate various aspects of plant growth and development have significantly reduced expression (Supplementary Table S3). For instance, expression of TF RPE5, which is responsible for the regulation of the gibberellin signaling pathway, was reduced eight fold; expression of nine different MADS-box TFs that control all key aspects of plant development, were two to 8 fold down-regulated; expression of auxin reponse factors, TFs from the auxin response factor (ARF) family, was reduced three-fold; expression of the TF IBH1, a negative regulator of cell elongation and plant development, was reduced ~six fold (Supplementary Table S3). Auxin signaling is critical to many plant growth and developmental processes from embryogenesis to senescence^12^,^13^. Thus, the overrepresentation of the GO term *protein dimerization activity* in the sets of down-regulated genes may be critical for the pleiotropic effect in RCPL5-silenced plants.

Although GO terms characterized as *transferase activity* were overrepresented in the up-regulated gene set, a majority of the genes in this category were down-regulated (Fig. 7a). Repressed genes in the category mostly encoded acyltransferases, proteins that are active in plant secondary metabolism pathways. These pathways contribute to the growth and development of plants but are not critical for their survival.

The VIGS-unique DEGs were further screened against the KEGG collection of databases to link transcriptomic information to metabolic pathways^14^. Among all KEGG pathways affected by RPC5L silencing, ribosome biogenesis and the biosynthesis of plant hormones zeatin and brassinosteroid were the most notable (Fig. 7b; Supplementary Figure S1).

Most of the DEGs participating in the ribosome biogenesis pathway were induced in VIGS plants, with the exception of the genes encoding proteins S3, S4, S13 and S14 (Supplementary Figure S1 and Supplementary Table S3). These proteins are located in the head and body regions of the small ribosomal subunit 40S^15^,^16^. In the ribosome, they are implicated in initiation of translation (S3), assembly and conformation of the 16S rRNA (S4) and ribosomal assembly. Protein S3, however, has extraribosomal functions in a number of cellular events, such as DNA repair, gene regulation and induction of apoptosis^17^.

There are 30 up-regulated genes encoding ribosomal proteins (RPs) of both large and small ribosomal subunits (Supplementary Table S3). Induction of RPs during virus infection was previously reported elsewhere^18^ and is possibly required for productive viral infection. However, since these DEGs are unique for VIGS plants only, silencing of the RPC5L has most likely caused their up-regulation, which might be needed for restructuring the protein synthesis system and ribosomal biogenesis under specific conditions of reduced RPC5L expression. The most up-regulated were RP genes for LP1, L18, L3, SAe and L23Ae proteins (Supplementary Table S3). The LP1 gene that is induced nearly 5-fold in VIGS plants, is part of the mobile structure that directs tRNA movement during translocation through the ribosome^19^. Ribosomal protein L18 was proposed to be a regulator of the double-stranded RNA (dsRNA)-activated protein kinase (PKR) activity that phosphorylates the alpha subunit of eukaryotic translation initiation factor 2 (eIF-2alpha). Activated PKR inhibits cell growth and induces apoptosis^20^.

Taken together, the activation of genes encoding components of the translational machinery may be explained by the plant trying to compensate for the ribosomal fraction that became dysfunctional due to the lack of 5S rRNA and tRNAs transcribed by RNAP III.

Three genes (Niben101Scf14562g00007.1, Niben101Scf06926g04001.1, Niben101Scf13934g00014.1) in the KEGG pathway Brassinosteroids Biosynthesis (BR) were down-regulated (Supplementary Figure S1). Repressed genes encode homologs of Tomato cytochrome P450 superfamily protein, castasterone 26-hydroxylase, and steroid 5 alpha reductase DET2. Mutants of *Arabidopsis* deficient in cytochrome P450 protein displayed dwarf phenotypes due to a lack of cell elongation^21^. Castasterone 26-hydroxylase modulates photomorphogenesis that is, plant development and growth mediated by light-spectrum. *Arabidopsis* with a mutant steroid 5 alpha reductase DET2 gene were small, dark-green dwarfs displaying pleiotropic defects in light-regulated development^22^. Overall, down regulation of key BR genes points to the fact that reduced activity of RPC5L in *N. benthamiana* plants critically affected BR physiological roles in normal plant growth and photomorphogenesis.

Five genes (Niben101Scf21960g01003.1, Niben101Scf02562g02012.1, Niben101Scf01386g00005.1, Niben101Scf01634g07023.1 and Niben101Scf04404g01009.1) in the KEGG pathway Zeatin Biosynthesis (Supplementary Figure S1) were significantly up-regulated. Zeatin is a plant hormone that promotes cell division in shoots, roots and lateral buds thus playing fundamental roles in growth and development^23^. Four of the genes encode cis-zeatin O-glucosyltransferase-like proteins that preferentially catalyze *O*-glucosylation of cis-Zeatin (cZ) and cZ-riboside^24^. Transgenic rice lines overexpressing several putative cZ-O-glucosyltransferases (cZOGTs) exhibited short-shoot phenotypes, delayed of leaf senescence, and decrease in crown root number^24^. The authors suggested that cZOGTs play important functions in cytokinin homeostasis and thus in the growth and development of rice. IPT2, adenylate isopentenyltransferase 5 catalyzes the initial step of cytokinin biosynthesis in higher plants, the formation of isopentenyladenosine 5′-monophosphate (iPMP) from AMP and dimethylallylpyrophosphate (DMAPP)^25^.

In addition to the overrepresented GO terms and KEGG annotations, we looked at the altered expression of individual genes and DEGs categories, whose portions within the set of DEGs were not significantly different from their distribution among all known *N. benthamiana* genes. Changes in their expression may be of significant interest due to their known biological roles or their important contribution to larger gene networks/pathways critical for plant development^26^.

Many genes in the not overrepresented GO term *peroxidase actitivity* (GO 0004601) were up-regulated (Supplementary Table S3). Among them were six genes encoding heme peroxidases, which are heme-containing enzymes that catalyze a number of oxidative reactions, including the reduction of H_2_O_2_ to water. For example, heme peroxidases 45 and 15 were up-regulated 15 and 51 fold, respectively. This not only implies that production of ROS (reactive oxygen species) is induced in response to stress, but also that the ROS-scavenging systems could be activated in VIGS plants. Presumably, in susceptible host-pathogen interactions such as PVX/*N. benthamiana*, the pathogen would elicit a low-amplitude, transient ROS accumulation^27^. Differences in gene expression accounted for this transient reaction should be negligible since VIGS plants are also PVX infected. DEGs were identified by comparisons between these two inoculation treatments, so the induction of heme peroxidases is likely to reflect either much higher rates of ROS production or their extensive detoxification in RPC5L- silenced plants.

Another not overrepresented GO category, *response to stress* (GO:0006952), included 47 DEGs (Supplementary Table S3). Induced MLO proteins (Powdery mildew locus O gene family) from a family of plant integral membrane proteins are thought to be involved in inhibition of cell death: their mutants have cell death control perturbations^28^. Six differentially expressed in VIGS plants major latex proteins (MLP), belong to the Bet v 1 family, also known as the pathogenesis related 10 (PR10)-like protein family^29^. The up-regulated MPL43 protein participates in abscisic acid (ABA) signal transduction, which is critical for plant growth and responses to abiotic stress. The universal stress protein (USP), induced nearly 5-fold in VIGS plants, mediates cellular responses to a wide variety of stresses^30^. Overall, a significant number of DEGs related to stress responses in VIGS plants may indicate that silencing of the RPC5L affected not only core functions of the RNAPIII that is, production of small housekeeping RNAs, but also had a broader influence on other processes, including defense responses.

#### Transcription factors differentially expressed in VIGS plants

Ninety eight transcription factors were differentially expressed in RPC5L-silenced plants (Supplementary Table S3). Many of them (37 TFs) are from the APR2/RF superfamily that regulates plant growth, development and responses to environmental stimuli^31^. WRKY, a large plant-specific family of TFs with numerous biological functions, including mediation of plant defense responses^32^, have a noticeable presence in the DEG TFs (15 TFs). WRKYs are also present as a part of hormone signaling net and can function up- and downstream of hormones^33^. Knock-down WRKY mutants have increased susceptibility to stresses^33^. Nine WRKY TFs were down-regulated in VIGS plants (Supplementary Table S3). Most of the MADS TFs, “transcription factors that shape the plants^34^” were down-regulated. In general, these data point to the attenuation of host immune system and reduced fitness of the VIGS plants as a consequence of the RPC5L silencing.

#### Ribosomal and transfer RNAs

Although RNA-sequencing method was based on the poly A selection, about 10.8% of the short reads belonged to the ribosomal transcripts. Digital expression showed that expression of 5S rRNA and tRNA (tyrosine) was reduced two-fold in VIGS plants (Supplementary Table S4). These numbers were even larger when quality control was performed with the same RNA samples by qPCR. Housekeeping non-protein-coding 5S rRNA and tRNA transcribed by RNAPIII are essential for translation. Two transcripts encoding U6 snRNA, a non-coding small nuclear RNA, were repressed (Supplementary Table S4). U6 snRNA is critical for pre-mRNA splicing^35^.

#### Chloroplast-related genes

A large group of genes (22 genes), whose protein products are functional components or enzymes located in the chloroplasts, was down-regulated in VIGS plants (Supplementary Table S3). Seven DEGs encoded proteins of Photosystem 1, an integral protein complex that mediates electron transfer from plastocyanin to ferredoxin^36^,^37^. One gene that is important for stability of the PSII complex was down-regulated. Expression of four genes from the carotenoid biosynthesis pathway (overrepresented KEGG pathway) was reduced (Supplementary Table S3). Chloroplast carotenoids act as membrane stabilizers and play major roles in photosynthesis, harvesting light and transferring the energy to chlorophylls^38^. The gene encoding protein Ycf2, previously reported to be indispensible for cell survival^39^, is down-regulated three-fold. Expression of heme oxygenase (HO, GO:0004392), a ubiquitous enzyme involved in heme degradation, was reduced two-fold. In higher plants it is found predominantly in chloroplast stroma and emerging roles for HO in plant defense against oxidative stress have been recently proposed^40^. Many proteins from the functional chloroplast are encoded in the nuclear genome and imported into the chloroplast after translation in the cytosol^41^. Geranyl diphosphate synthase (GDS), that is presumably targeted to plastids and required for consolidation of isopentenyl pyrophosphate (IPP) and dimethylallyl pyrophosphate (DMAPP) to geranyl diphosphate (GPP), was found to be down-regulated more than fivefold in VIGS plants. IPP and DMAPP are the first precursors for the synthesis of various terpenoids employed by plants for a variety of basic functions in growth, defense and development^42^. Virus-induced silencing of the gene in tomato and silencing using RNAi constructs in Arabidopsis resulted in dwarfed phenotypes^43^. Three transcripts of geranylgeranyl diphosphate synthase (GGDS) presumably coding isozymes were 2 to 5 fold down-regulated. GGDS participates in biosynthesis of geranylgeranyl diphosphate (GGPP), the precursor for the biosynthesis of gibberellins, carotenoids, chlorophylls, isoprenoid quinones, and geranylgeranylated proteins in plants^44^.

#### Cell death-related genes

The gene encoding Metacaspase 1 (Gene ID Niben101Scf07585g00003.1; Metacaspase 1, Caspase-like domain) was induced more than three-fold. Metacaspases are mediators of program cell death in plants^45^. Two cyclin-D-binding Myb-like transcription factors were activated as much as 25–29 fold (Gen IDs Niben101Scf06736g08010.1 and Niben101Scf05998g01004.1, respectively; Supplementary Tables S2 and S4). Human DMTF1 cyclin D binding Myb-like transcription factor 1 is involved in tumor suppression and induction of apoptosis^46^. The DMTF1 is activated by oncogenic Ras signaling and displays tumor suppressing activity through induction of the Arf-p53 pathway, which causes cell-cycle arrest or apoptosis^46^.

#### Other interesting genes affected by VIGS

Squamosa-promoter binding protein (SBP, Gene ID Niben101Scf00878g04013.1) a transcription factor involved in the control of early flower development and floral organogenesis^47^ was down-regulated over 12 fold (Supplementary Table S2). Expression of several cell cycle-related genes from cyclin family that controls cell proliferation in plants was inhibited two to four times in VIGS plants (Gene IDs Niben101Scf29276g00001.1, Niben101Scf01063g09014.1, Niben101Scf07793g02024.1 and Niben101Scf01397g00020.1). A member of the Snakin/GASA family of pathogenesis-related proteins was down-regulated more than ten-fold (Gene ID Niben101Scf01578g00012.1). Snaking proteins are thought to be a critical component of innate immunity participating in hormonal signaling pathways^48^,^49^.

### Confirmation of digital expression data by qPCR

To further validate digital expression data, qPCR was performed with 61 selected genes classified as differentially expressed based on the analysis of the transcriptome. qPCR of the DEGs presumably associated with RPC5L silencing showed a strong correlation with the transcriptomic data (Pearson correlation coefficient r = 0.945729; Supplementary Table S4).

## Discussion

Although RNAP III is known to transcribe housekeeping RNAs such as rRNA, tRNA, U6 snRNA, the RNA subunit of RNase P, 7SL RNA and other short RNAs, the functional diversity of the transcribed genes may be wider^50^. The RPC5L is one of the 17 widely conserved RNAP III subunits and its exact functional role in the transcription is poorly known. It has been proposed that in yeast *S. cerevisiae* the C37 subunit (a counterpart of the RPC5L) is structurally related to the basal transcription factor TFIIF, which is involved in the initiation and elongation phase of transcription^51^. In the absence of the subassembly consisting of the C37 and C53 (another RNAP III subunit), transcription fails to proceed beyond the transcriptional start^52^. To our knowledge, there are no published data addressing a role and consequences of a loss of function of the RPC5L in Plantae. The vast majority of accumulated information on the RNAP III and its subunits has derived from human and yeast sources.

In this work, we report that silencing of the putative RPC5-like subunit of the RNAP III in a model species *Nicotiana benthamiana* affected vital functions critical for normal plant growth, development and response to the environment. Virus-induced gene silencing of the RPC5L transcripts caused pleiotropic defects including but not limited to severe dwarfing appearance, vein clearing, chlorosis, shorter internodes, abnormal leaf shape and deformed floral phenotypes.

Transriptomic analysis of VIGS plants demonstrated that phenotypic abnormalities are primarily related to the several main events occurring in the RPC5L-silenced plants: reduced expression of genes controlling protein dimerization activity, down-regulation of genes in the brassinosteroids biosynthesis pathway, induced expression of genes in the zeatin biosynthesis pathway and down-regulation of rRNA and chloroplast-related genes. Protein dimerization controls diverse events on both organismal and cellular levels, including signal transduction from the cell surface to nucleus^53^. Brassinosteroids (BR) are plant hormones that promote stem elongation, cell division, vascular differentiation, etiolation, and reproductive development^54^. Brassinosteroids also modulate photomorphogenesis, a response to light spectrum. Zeatin is a plant hormone that among other effects can promote growth of lateral buds. Therefore, up-regulation of genes in zeatin pathways might be responsible for bushier phenotypes of the VIGS plants. Pleiotropic effects of RPC5L silencing can also be attributed to the disruption of the core RNAPIII functions i.e. synthesis of ncRNAs. Indeed, expression of rRNA and tyrosine tRNA was reduced in VIGS plants. 5S rRNA is associated with large subunit of cytozolic riobosomes^50^ and is the smallest of the RNA components of the ribosome that binds ribosomal proteins prior to its assembly into the ribosome^54^. Although the exact functions of 5S rRNA are still largely unknown, it has been proposed that it is critical for tRNA binding to the ribosome, for structural connections between different regions of the ribosome and for coordination of translation^55^. Inhibition of 5S rRNA transcription would adversely effect these general functions, thus disrupting ribosomal architecture and protein synthesis. Whether it non-specifically affected all translational machinery or only its individual steps and related proteins encoded by DE genes described in this work, remains unknown. Down-regulation of tyrosine tRNA may potentially imply that signaling pathways are affected. In animals, tyrosine is a component of the receptor tyrosine kinases, which are key regulators of cellular proliferation and differentiation^56^. In plants, it was recently demonstrated that tyrosine phosphorylation is associated with key enzymes in the brassinosteroid signaling pathway^57^.

VIGS plants with silenced RPC5L had distinct vein clearing/chlorosis symptoms (Fig. 2), which could be due to the suppression of genes encoding essential chloroplast proteins (Supplementary Table S3). For instance, depleted activities of the photosystems could affect plants at multiple levels related to their roles in oxidation of H_2_O, NADP reduction and synthesis of ATP^37^,^58^. Inhibition of genes from carotenoid biosynthesis may interconnect with overlapping metabolic pathways, plastid differentiation and with developmental and environmental responses^38^. Reduction of geranyl diphosphate synthase expression in VIGS plants could be of critical importance for generation of many post-silencing effects, including dwarfing, via implications on biosynthesis of gibberellins^43^.

Activation of tentative apoptosis-related genes (metacaspase 1 and two cyclin-D-binding Myb-like transcription factors) might not be a random aftermath of the RPC5L silencing, especially considering occasional accumulation of dead cells in the leaves of VIGS plants (Fig. 4). Speculatively, these observations could be linked to the engagement of defense mechanisms in response to the broad effects of RPC5L silencing. However, since no information on the DMTF1 orthologs in plants is currently available and as a result the annotation is apparently based on the presence of Myb domain, activation of the genes could be associated with a number of other processes regulated by the MYB family of proteins^59^.

The association of many DEGs in VIGS plants with defense pathways (Supplementary Table S3) and the down-regulation of a significant portion of them indicates that the biological functions of RCP5L overlap with and/or are an important component of the plant immune system.

**Figure 8:**
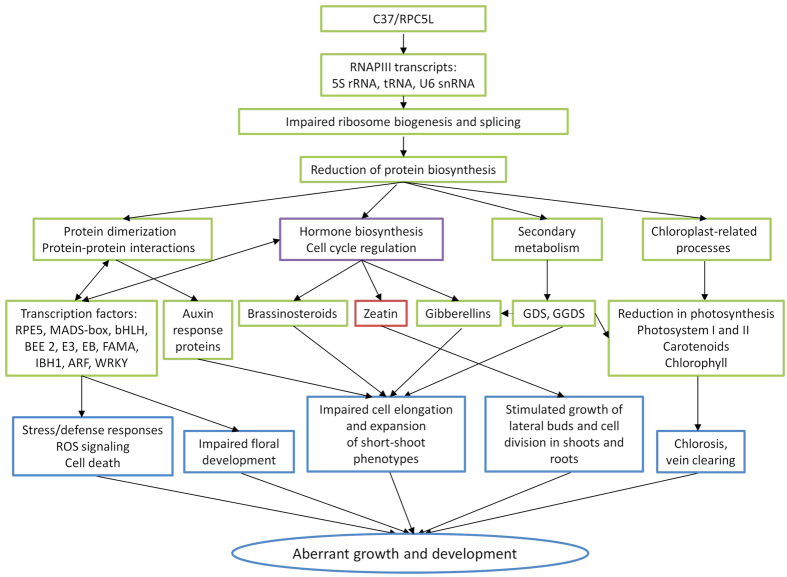
Schematic representation of the tentative biological roles of RCP5L subunit of RNAPIII, deduced from the results of VIGS assay and transcriptomic analysis of *N. benthamiana* plants with silenced RCP5L gene. Green outline: down-regulated processes; red outline: up-regulated processes; purple outline: up-and down-regulated processes, blue outline: phenotypic effects.

Figure 8 schematically represents tentative biological roles of RPC5L subunit of RNAPIII, deduced from the results of VIGS assay and transcriptomic analysis of *N. benthamiana* plants with a silenced RPC5L gene.

In summary, silencing of the RPC5L resulted in distinct phenotypic abnormalities and had a pronounced effect on a broad range of cellular processes. This implies that the normal functioning of the subunit is altogether critical for plant development and survival. Recently, Johnson *et al*.^60^ demonstrated that a loss-of-function mutant of *Arabidopsis* RNAPIII subunit *nrpc7-1* displayed multiple phenotypic defects including dwarf morphology, stunted siliques and serrated leaves^60^. Mutation also resulted in global changes in RNA levels. It is, therefore, remains a question if the observed changes in gene expression and relevant phenotypic deviations in our VIGS plants were specifically associated with underexpressed RPC5L gene or if the knock-down of any other RNAPIII subunit would cause similar consequences. Importantly, however, the vast majority of RNAs with altered expression in RPC5L-silenced plants were not transcribed by RNAPIII, which resonates with the study of Johnson *et al*.^60^. Among affected genes were those seemingly unrelated to the widely accepted biological roles of RNAPIII, most notably from defense and stress-associated pathways.

## Materials and Methods

### Vector construction

Primers for amplification of the *N. benthamiana* ortholog of At5G49530 were designed based on the *Nicotiana* EST sequence obtained through NCBI tblastn search against the translated gene product of At5G49530. The RPC5L fragment was amplified from healthy *N. bentamiana* leaf tissues using primers LN191 and LN192 (Supplementary Table S1). The *Eco*RV restriction site was introduced in both forward and reverse primers to facilitate subsequent cloning into *Eco*RV- linearized PVX vector pP2C2S^61^ (obtained from the Sainsbury Laboratory, Norwich, UK). The amplified 446-nt long PCR product was first directly cloned into the TOPO 2 vector (ThermoFisher Scientific) followed by subcloning into the pP2C2S. The *Tobacco rattle virus* (TRV)-based vector, obtained from Arabidopsis Biological Resource Center, Ohio State University, was also used in VIGS experiments^62^. The same RPC5L fragment was cloned into *Eco*RI-linearized plasmid pYL156 (a clone of the TRV RNA 2). The integrity of all clones was verified by automated sequencing.

### Plant growth and inoculation of plants

*N. benthamiana* (*Nicotiana benthamiana* L.) plants were grown in the containment greenhouse facilities at the Molecular Plant Pathology Laboratory, USDA/ARS/BARC, Beltsville, MD, USA, with supplemental lamp illumination and a photoperiod of 16 hours. Fully expanded leaves of *N. benthamiana* were inoculated with capped RNA transcripts generated from the SpeI-linearized pP2C2S vector using Ambion’s T7 mMessage Machine kit as advised by manufacturer (ThermoFisher Scientific). Plants were inoculated with recombinant TRV vector essentially as described in Liu *et al*.^8^.

Tissues from five non-inoculated (systemic) leaves from each of the four plants per variant (uninoculated control, PVX and PVX/RPC5L) were harvested after symptoms were fully developed (~21 dpi), snap frozen in liquid nitrogen, and stored at 80 °C until RNA extraction.

### RNA extraction, primer design, cloning and quantitative real-time PCR

Total RNA was extracted with a FastRNA Pro Green Kit (MP Biomedicals), and subsequently purified using RNA easy columns (RNeasy Mini Kit, Qiagen Inc.). Amplification was conducted with a Rotor Gene Q real time PCR cycler (Qiagen) using Rotor-Gene SYBR^®^ Green PCR kit (Qiagen) in four biological and two technical replicas using the following parameters: 95 °C/10 min (one cycle), 95 °C/10 s, and 60 °C/45 s (40 cycles). cDNA for qPCRs was made with random hexamers from the same RNA samples that were used for RNA-sequencing. *N. benthamiana* elongation factor-1α (*NbEF-1a*, accession AY206004) was used as internal reference transcripts in all real-time PCR experiments (Supplementary Table S1). The specificity of all amplifications was confirmed by single-peak melting curves. The Delta Delta C (T) method (2^−ΔΔ*C*^^T^) was used for analysis of relative expression^63^. To obtain a final ratio for any given gene, an average and a standard deviation (SD) for all biological replicates were calculated.

### RNA-sequencing and gene expression profiling

RNA-sequencing was performed by the Next Generation Sequencing Center, Johns Hopkins University (Baltimore, MD, USA) for a fee using the Illumina HiSeq 2000 system. There were four replicates for each experimental condition and for the untreated control. cDNA libraries were generated using a poly A selection method. The strand-specific paired-end-reads were mapped onto the available draft transcriptome sequence of *N. benthamiana* (permission to use the *Nicotiana benthamiana* genome sequence was obtained from Drs. G. Martin and A. Bombarely) using the strand specific option forward and reverse of the RNAseq analysis module in the CLC Genomic Workbench 8.0.2 software (Qiagene). The DESeq 2 package from Bioconductor was used to estimate sample quality (PCA) and expression level of the genes. Genes with fold change more than 2 and false discovery rate (FDR) less than 0.001 and number of mapped reads more than 50 were counted as a differentially expressed.

Because there is no KEGG map for *N. benthamiana*, we used BlastKOALA tool to find homologs in tomato followed by using Search&Color Pathway to map *N. benthamiana* transcripts to Tomato KEGG pathways. For overrepresentation analysis of GO terms and for KEGG overrepresentation, AgriGO web service and Exact Fisher test were used, respectively, as described previously^26^.

### Measurement of the photosynthesis rates

Photosynthesis rates were measured based on the uptake of CO_2_ using a Portable Photosynthesis System LI-6400XT as described by manufacturer (LI-COR, Nebraska USA.

## Additional Information

**How to cite this article**: Nemchinov, L. G. *et al*. Virus-induced gene silencing of the RPC5-like subunit of RNA polymerase III caused pleiotropic effects in *Nicotiana benthamiana*. *Sci. Rep.* **6**, 27785; doi: 10.1038/srep27785 (2016).

## Supplementary Material

Supplementary Figure S1

Supplementary Table S1

Supplementary Table S2

Supplementary Table S3

Supplementary Table S4

## Figures and Tables

**Table 1:** Average percentage of the total reads mapped to the reference transcriptome of *Nicotiana benthamiana* and reference genome of *Potato virus X* from the four control, four PVX-WT and four VIGS libraries.

Variant	Nb sense %	Nb antisense %	PVX %
Control	64.3	9.1	0.0
PVX-WT	50.1	8.6	10.0
VIGS	64.0	6.8	3.2

Nb sense and Nb antisense: sequenced reads from sense and antisense orientation, respectively, mapped to *N. benthamiana* transcriptome. PVX: sequenced reads mapped to the reference genome of *Potato virus X*.
