# Supplementary material for: Virus-induced gene silencing of the RPC5-like subunit of RNA polymerase III caused pleiotropic effects in Nicotiana benthamiana
Source: Sci Rep. 2016 Jun 10;6:27785. doi: 10.1038/srep27785 (PMC4901293; doi:10.1038/srep27785)
Supplement: Supplementary Figure S1 [file srep27785-s1.pdf]

Lev G. Nemchinov, Alexander M. Boutanaev and Olga A. Postnikova. Virus-induced gene silencing of RPC5-like subunit of RNA polymerase III caused pleiotropic effects in Nicotiana benthamiana.

Supplementary Figure S1

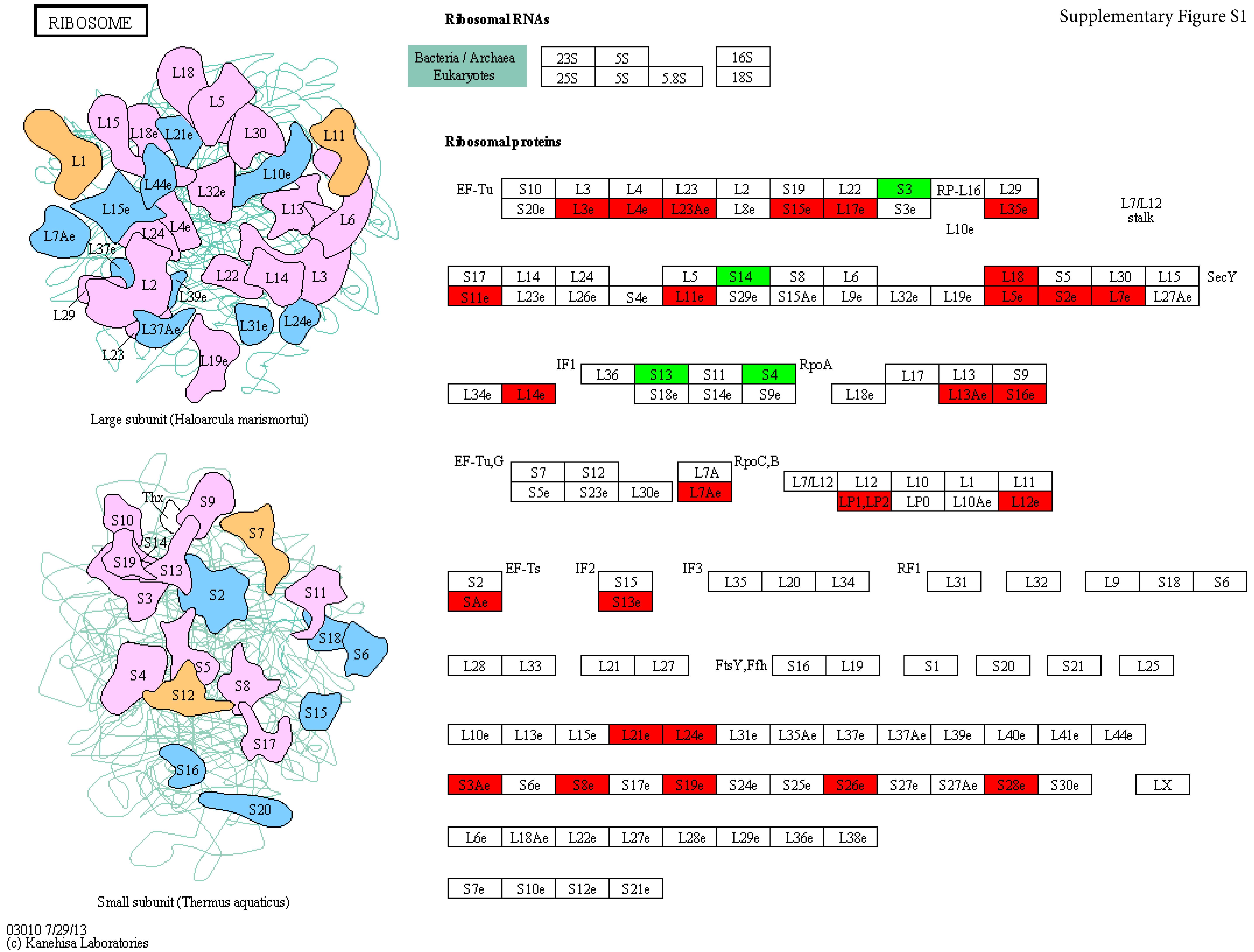

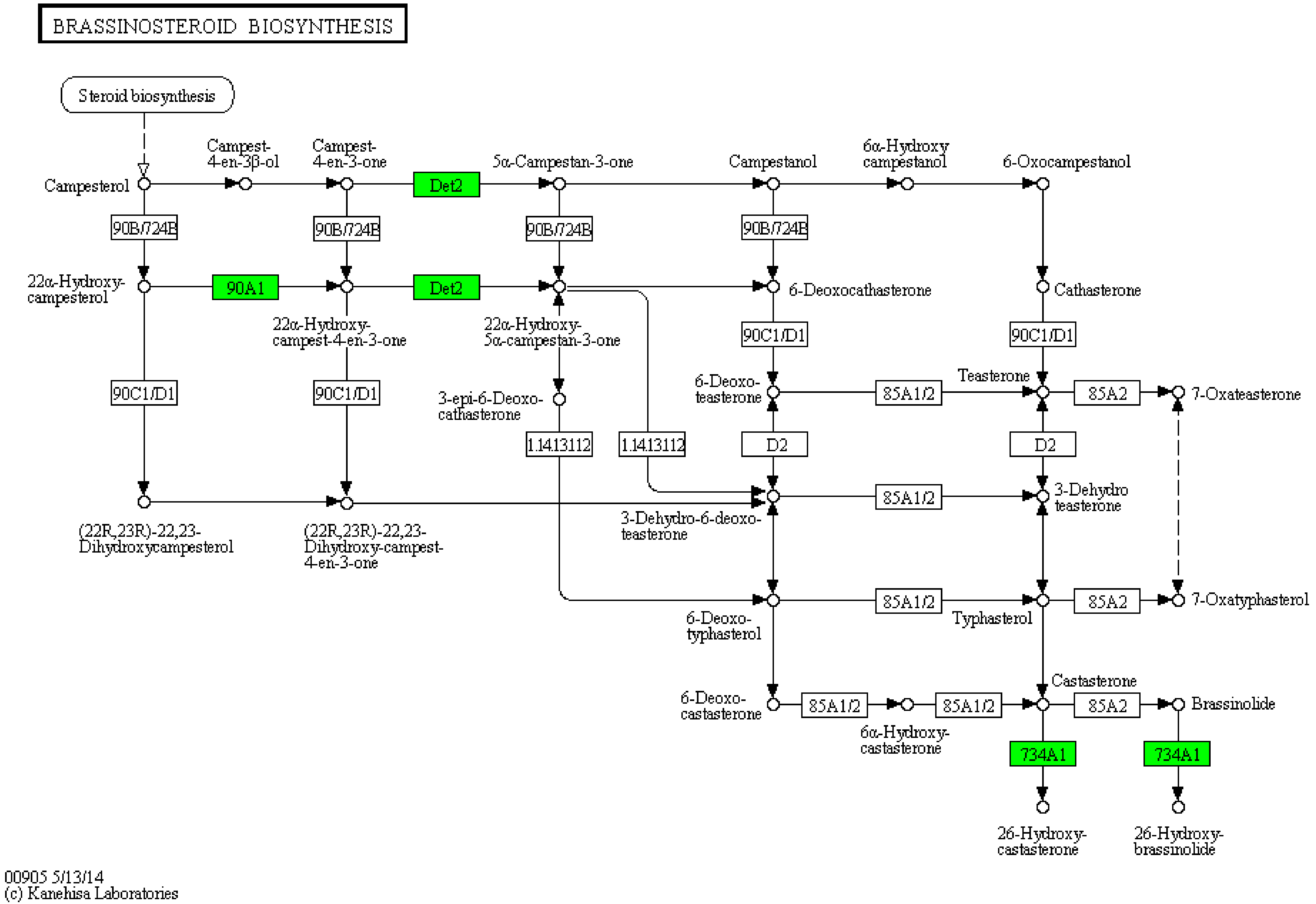
BRASSINOSTEROID BIOSYNTHESIS
Steroid biosynthesis
Campesterol
Campest-4-en-3β-ol
Campest-4-en-3-one
Det2
5α-Campestan-3-one
Campestanol
6α-Hydroxy campestanol
6-Oxocampestanol
90B/724B
90B/724B
90B/724B
90B/724B
22α-Hydroxy-campesterol
90A1
Det2
6-Deoxocathasterone
Cathasterone
22α-Hydroxy-campest-4-en-3-one
22α-Hydroxy-5α-campestan-3-one
90C1/D1
90C1/D1
90C1/D1
90C1/D1
3-epi-6-Deoxo-cathasterone
6-Deoxo-teasterone
85A1/2
Teasterone
85A2
7-Oxateasterone
1.14.13.112
1.14.13.112
D2
D2
3-Dehydro teasterone
85A1/2
(22R,23R)-22,23-Dihydroxycampesterol
(22R,23R)-22,23-Dihydroxy-campest-4-en-3-one
3-Dehydro-6-deoxo-teasterone
85A1/2
Typhasterol
85A2
7-Oxatyphasterol
6-Deoxo-typhasterol
6-Deoxo-castasterone
85A1/2
6α-Hydroxy-castasterone
85A1/2
Castasterone
85A2
Brassinolide
734A1
734A1
26-Hydroxy-castasterone
26-Hydroxy-brassinolide
00905 5/13/14
(c) Kanehisa Laboratories

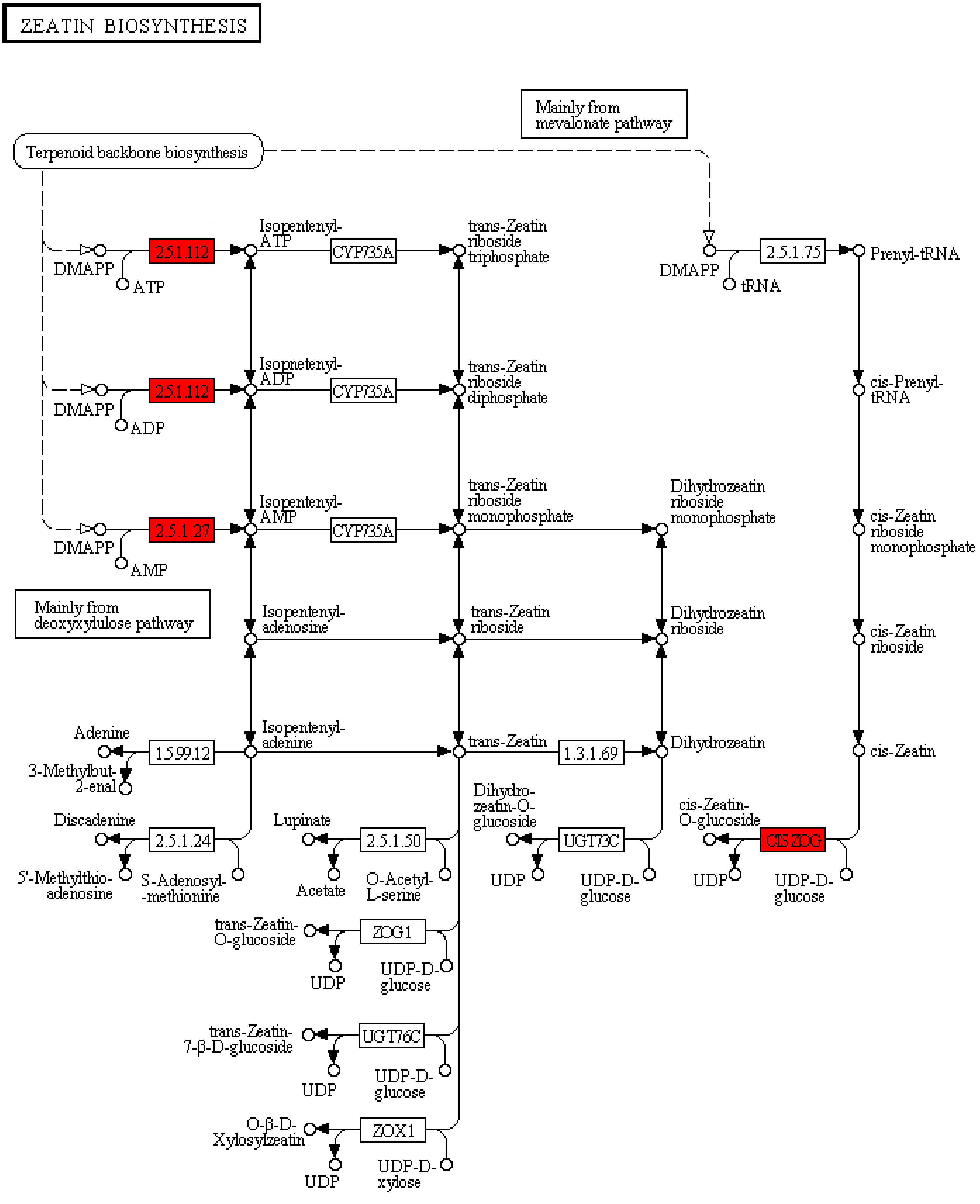

00908 5/23/14
(c) Kanehisa Laboratories
